# Areal bone mineral density is not associated with femoral stem subsidence in patients younger than 70 years undergoing total hip arthroplasty

**DOI:** 10.1007/s00402-023-05137-w

**Published:** 2023-12-07

**Authors:** Tim Rolvien, Maximilian Lenard Thiessen, Christoph Kolja Boese, Ulrich Bechler, André Strahl, Frank Timo Beil, Christian Ries

**Affiliations:** Division of Orthopaedics, Department of Trauma and Orthopaedic Surgery, University Medical Center Hamburg-Eppendorf, Martinistraße 52, 20246 Hamburg, Germany

**Keywords:** THA, Femur, Subsidence, Bone quality, DXA

## Abstract

**Introduction:**

Femoral stem subsidence is a known complication after uncemented total hip arthroplasty (THA). The purpose of this study was to determine the frequency of osteoporosis and to investigate the relationship between areal bone mineral density (aBMD) and subsidence in a cohort of patients younger than 70 years.

**Methods:**

One hundred consecutive patients (age 60 ± 6 years; 52 female, 48 male) undergoing uncemented THA using a collarless press fit femoral stem were retrospectively reviewed. Dual-energy X-ray absorptiometry (DXA) was performed preoperatively at the proximal femur and lumbar spine, and if not feasible at these sites, at the distal radius. DXA results were compared to a cohort of 100 patients ≥ 70 years scheduled for cemented THA. Age, sex, and body mass index (BMI), canal flare index (CFI), and canal fill ratio (CFR) were assessed. Analysis of stem subsidence and migration was performed on standardized, calibrated radiographs obtained postoperatively and at follow-up.

**Results:**

The frequency of osteoporosis was considerably lower in the study cohort compared to patients ≥ 70 years (7% vs. 19%, p = 0.02). Illustrated by the high CFR (mean 96 ± 4%) in the mid-stem region, a sufficient press fit was achieved. After a mean follow-up of 7.4 months, the mean stem subsidence was 0.9 ± 0.9 mm. Only two patients had subsidence greater than 3 mm, one of whom was morbidly obese and the other diagnosed with severe osteoporosis. There were no correlations between any of the parameters (CFI, CFR, age, sex, BMI) and femoral stem subsidence. In addition, aBMD T-scores showed no correlations with subsidence.

**Conclusion:**

aBMD by DXA does not appear to be associated with stem subsidence in patients younger than 70 years and with adequate press fit.

## Introduction

Total hip arthroplasty (THA) is a well-established treatment of end-stage osteoarthritis (OA) of the hip [1]. The number of procedures is expected to further increase due to demographic changes in the next decades [2]. Reduced bone quality (i.e., osteopenia or osteoporosis) is common in older patients with OA [3–5]. However, optimal stem fixation in elderly patients (i.e., cemented vs. uncemented) in THA is still under debate. Registry data have shown an increased risk of early failure for uncemented stems in patients older than 75 years of age or with a body mass index (BMI) > 30 kg/m^2^ [6]. Furthermore, previous studies have documented osteoporosis in approximately 18% of the patients ≥ 70 years who were scheduled for THA [3], and more than 40% had osteopenia.

Adequate bone quality is decisive for osseointegration and prevention of implant-related complications [7]. Therefore, patients should be screened for reduced areal bone mineral density (aBMD) by dual-energy X-ray absorptiometry (DXA) prior to THA, according to current recommendations [8, 9]. Based on the known risk factors for complications and early implant failure, elderly patients should possibly undergo cemented stem fixation as a gold standard. Nevertheless, no generally accepted cutoff or recommendation regarding an age limit concerning uncemented stem fixation exist.

The present study aimed to analyze bone quality by DXA in patients < 70 years who underwent uncemented, collarless THA. We hypothesized that these patients are not as likely to have decreased aBMD as older patients but low aBMD may be associated with increased risk of stem subsidence. Additionally, we investigated potential influencing anatomical and individual parameters on uncemented stem fixation. Specifically, the aims of the present study were: (1) analysis and comparison of bone density by DXA in patients < 70 vs. ≥ 70 years scheduled for THA due to end-stage OA, (2) characterization of stem subsidence and migration in uncemented, collarless primary THA < 70 years, and (3) investigation of potential influencing anatomic (e.g., canal flare index (CFI), canal fill ratio (CFR)), demographic (e.g., age, sex, BMI), and densitometric parameters on stem subsidence.

## Methods

### Study cohort

A retrospective review of 100 consecutive patients (52 female, 48 male) with available DXA measurements who underwent uncemented THA using a collarless, dual tapered-wedge hydroxyapatite-coated press fit femoral stem (Avenir^®^ Hip System Ti-6Al-4V, Zimmer, Warsaw, IN, USA) was carried out. THA was performed by experienced senior surgeons via a posterior approach. A metaphyseal stem fixation was aimed for. Full weight bearing was allowed directly after surgery. Patients with primary or secondary end-stage OA were included. Malignancy of the femur or pelvis, posttraumatic femoral deformation, and severe hip dysplasia (Hartofilakidis type B or C) served as exclusion criteria. For comparison of aBMD values, a control cohort of 100 patients ≥ 70 years undergoing cemented THA (Müller™ straight stem, Zimmer) was included. This study was approved by the local ethics committee (2021-300036-WF) and followed the statements of the most recent version of the Declaration of Helsinki.

### Anatomical parameters and subsidence

Postoperative (within one week after surgery) and follow-up radiographs (minimum eight weeks) were digitally analyzed using a Picture Archiving and Communication System (PACS) (IMPAX EE, Agfa HealthCare GmbH, Germany) according to Ries et al. [10]. Analyses of anatomical parameters (CFI, CFR), stem subsidence and migration (stem angulation) were performed on standardized, calibrated, standing anteroposterior radiographs in all patients who underwent uncemented stem fixation. Calibration was performed by measuring the 32 mm ceramic femoral head used. Severity of stem subsidence was graduated as previously described by Al-Najjim et al. [11].

### Dual energy X-ray absorptiometry (DXA)

Areal bone mineral density (aBMD) was assessed by Dual energy X-ray absorptiometry (DXA, Lunar Prodigy enCore 2007, GE Healthcare; Madison, WI, USA) performed at the left and right proximal femur as well as the lumbar spine (L1–L4). At the proximal femur contralateral to the operated side, no measurement was performed if a prosthesis was in situ. At the lumbar spine, measurements were excluded if advanced degeneration was present. In 13 patients with morbid obesity (BMI > 35 kg/m^2^), DXA was performed at the distal radius. All measurements were performed within four weeks before surgery. T-scores expressing aBMD standard deviations for young, sex-matched healthy adults were generated using the manufacturer's software. Based on the T-score, osteoporosis and osteopenia were diagnosed according to World Health Organization (WHO) guidelines (i.e., normal T-score > − 1.0, osteopenia T-score > − 2.5 ≤ − 1.0, osteoporosis T-score ≤ − 2.5) [12]. As aBMD may be overestimated in the osteoarthritic hip (as well as in the lumbar spine), we also used the contralateral hip T-score as well as the lowest T-score of any of the measured sites for further analysis.

### Statistical analysis

GraphPad Prism^®^ (version 9.0, GraphPad Software, La Jolla, CA) was used for statistical analysis. Data are expressed as mean ± standard deviation (SD). To compare two groups, the student’s t-test was used for normally distributed data and the Mann–Whitney-U test for non-parametric data. Associations were analyzed by Pearson’s correlation analysis. Furthermore, the relative risk of stem subsidence was calculated based on the aBMD T-score cutoffs and the individual risks were compared using Fisher’s exact test. Statistical significance was set to a p-value of 0.05. We calculated the effect size using G*Power [13] based on the results of a previous study [14], which showed that patients with low BMD have higher stem subsidence during the first 3 months after surgery than did those with normal BMD (difference = 0.6, 95% confidence interval (CI): 0.1–1.1; p = 0.03). These results correspond to an effect size of d_Cohen_ = 0.824, which is equivalent to an effect size of f^2^ = 0.17. With an alpha level set at 0.05, a power of 0.95, and an expected medium effect (f^2^ = 0.17), a required sample size of n = 79 was calculated to investigate the influence of aBMD on subsidence (linear regression analysis). The intraclass correlation coefficient (ICC) was used to assess interrater reliability. A two-way mixed effects model with absolute agreement was used. Subsidence measurements were performed by two investigators in 20 patients, yielding an ICC of 0.99 (95% CI 0.974–0.996). Based on the high agreement, the measurements of one investigator were used for the analysis of the complete data set.

## Results

### Lower prevalence of osteoporosis in patients < 70 vs. ≥ 70 years

**Fig. 1:**
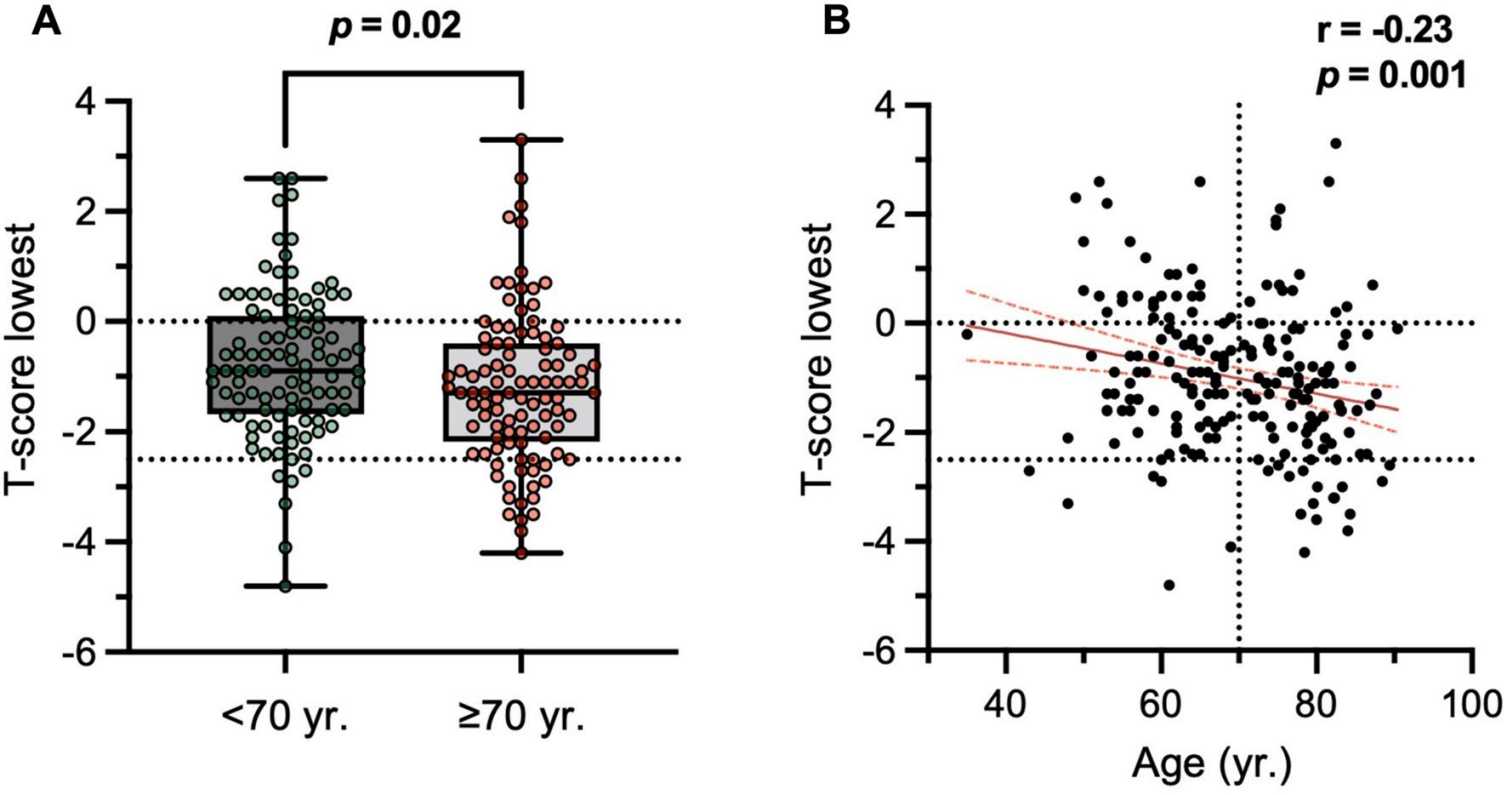
Age-related changes of DXA outcomes. **A** Comparison of DXA BMD T-scores in the study cohort (uncemented THA, < 70 years) and the control cohort (cemented THA, ≥ 70 years). **B** Correlation analyses of age and T-scores. The 95% confidence bands of the best fit line in a simple linear regression analysis are shown. Exact r- and p-values are presented above the panels

Compared with the control cohort of older patients (≥ 70 years), patients < 70 years were equal in sex ratio but had higher BMI values (Table 1). aBMD T-scores assessed by DXA at both femora were lower than in the aged group, whereas spinal T-scores and T-scores of the affected hip were not different. When considering the lowest T-score of any measurement site, higher values were also observed in patients < 70 years than in those ≥ 70 years (Fig. 1A). There was also a weak negative correlation with age in the overall cohort (Fig. 1B). Osteoporosis and osteopenia were detected in 7% and 39% of the patients < 70 years, respectively.

**Table 1:** Overview of the study cohort (patients < 70 years undergoing uncemented THA) compared to a control cohort of elderly patients ≥ 70 years undergoing cemented THA

Parameter	Uncemented (< 70 yr, n = 100)		Cemented (≥ 70 yr, n = 100)		*p*
	Mean ± SD	Min–Max	Mean ± SD	Min–Max	
Demographics					
Age (yr)	60.4 ± 6.3	35.0–69.0	78.7 ± 4.7	70.7–90.4	** < 0.0001**
Sex (f:m)	52:48		58:42		0.39
Weight (kg)	92.3 ± 22.3	49.0–142.0	77.4 ± 13.7	50.0–110.0	** < 0.0001**
Height (m)	1.74 ± 0.10	1.50–2.07	1.67 ± 0.08	1.50–1.84	** < 0.0001**
BMI (kg/m^2^)	30.2 ± 6.0	18.8–48.0	27.5 ± 4.1	20.0–35.9	**0.001**
DXA					
Left femoral T-score	− 0.5 ± 1.2	− 4.8–2.6	− 0.8 ± 1.3	− 4.2–3.3	**0.07**
Right femoral T-score	− 0.6 ± 1.1	− 2.8–2.7	− 0.9 ± 1.3	− 3.3–3.3	**0.02**
Spinal T-score	− 0.4 ± 1.5	− 4.7–4.5	− 0.2 ± 1.9	− 3.8–5.6	0.61
T-score lowest	− 0.8 ± 1.3	− 4.8–2.6	− 1.3 ± 1.4	− 4.2–3.3	**0.02**
T-score aff. hip	− 1.0 ± 1.1	− 4.8–2.2	− 0.8 ± 1.3	− 4.2–3.3	0.51
Normal BMD	54%		37%		**0.02**
Osteopenia (< − 1.0)	39%		44%		0.57
Osteoporosis (≤ − 2.5)	7%		19%		**0.02**

Bold indicates significant differences (*p* < 0.05)

### Femoral stem subsidence and correlation with traditional anatomic parameters

After a mean follow-up of 7.4 months, the mean stem subsidence was 0.9 ± 0.9 mm. No surgical stem revision was performed in any of the cases during the follow-up period. A subsidence of < 3 mm was recorded in 98 patients, while only 2 patients had a subsidence greater than 3 mm (Table 2). In total, subsidence greater than 2 mm was observed in ten patients. Evaluation of the CFI indicated a “stovepipe”, normal, and “champagne-fluted” femur anatomy in 24%, 72%, and 4% of the patients, respectively. Illustrated by the high CFR (mean 96 + 4%) in the mid-stem region, a sufficient press fit was achieved. The follow-up radiographs showed that the deviation from the initial stem angulation did not exceed 3°, except for one patient who also experienced more than 5 mm of subsidence. No significant correlations were found between any of the traditional anatomic parameters and femoral stem subsidence, including CFI (r = − 0.01, p = 0.89), CFR (r = − 0.04, p = 0.70), age (r = 0.17, p = 0.10), sex (r = 0.06, p = 0.55), and BMI (r = 0.03, p = 0.80) (Table 3).

**Table 2:** Results of the subsidence analysis as well as anatomic parameters measured in pre- and postoperative standing anteroposterior radiographs of the hip

Parameter	Mean ± SD	Min–max
Subsidence (mm)	0.9 ± 0.9	0.0–5.1
Group I (< 3 mm)	98%	
Group II (3–5 mm)	1%	
Group III (> 5 mm)	1%	
Canal Flare Index	3.5 ± 0.6	2.3–5.2
"Stovepipe" (< 3.0)	24%	
"Normal" (3.0–4.7)	72%	
"Champagne-fluted" (> 4.7)	4%	
Canal fill ratio (%)		
Stem shoulder	75 ± 11	52–99
Mid stem	96 ± 4	72–100
2 cm above stem tip	90 ± 8	62–100
Stem angulation (°)	1.2 ± 0.8	− 0.9–2.9
Dev. initial stem ang. (°)	0.0 ± 0.5	− 3.4–1.1

Varus and valgus deviation from initial stem angulation are reported as positive and negative values

**Table 3:** Correlations between anatomic or demographic parameters and stem subsidence

Parameter	r	*p*
CFI	− 0.01	0.89
CFR	− 0.04	0.70
Age	0.17	0.10
Sex	0.06	0.55
BMI	0.03	0.80

Correlation coefficients r and p-values are presented. Sex was considered as a bivariate variable (1: male, 2: female)

*CFI* canal flare index, *CFR* canal fill ratio, *BMI* body mass index

### DXA and subsidence

**Fig. 2:**
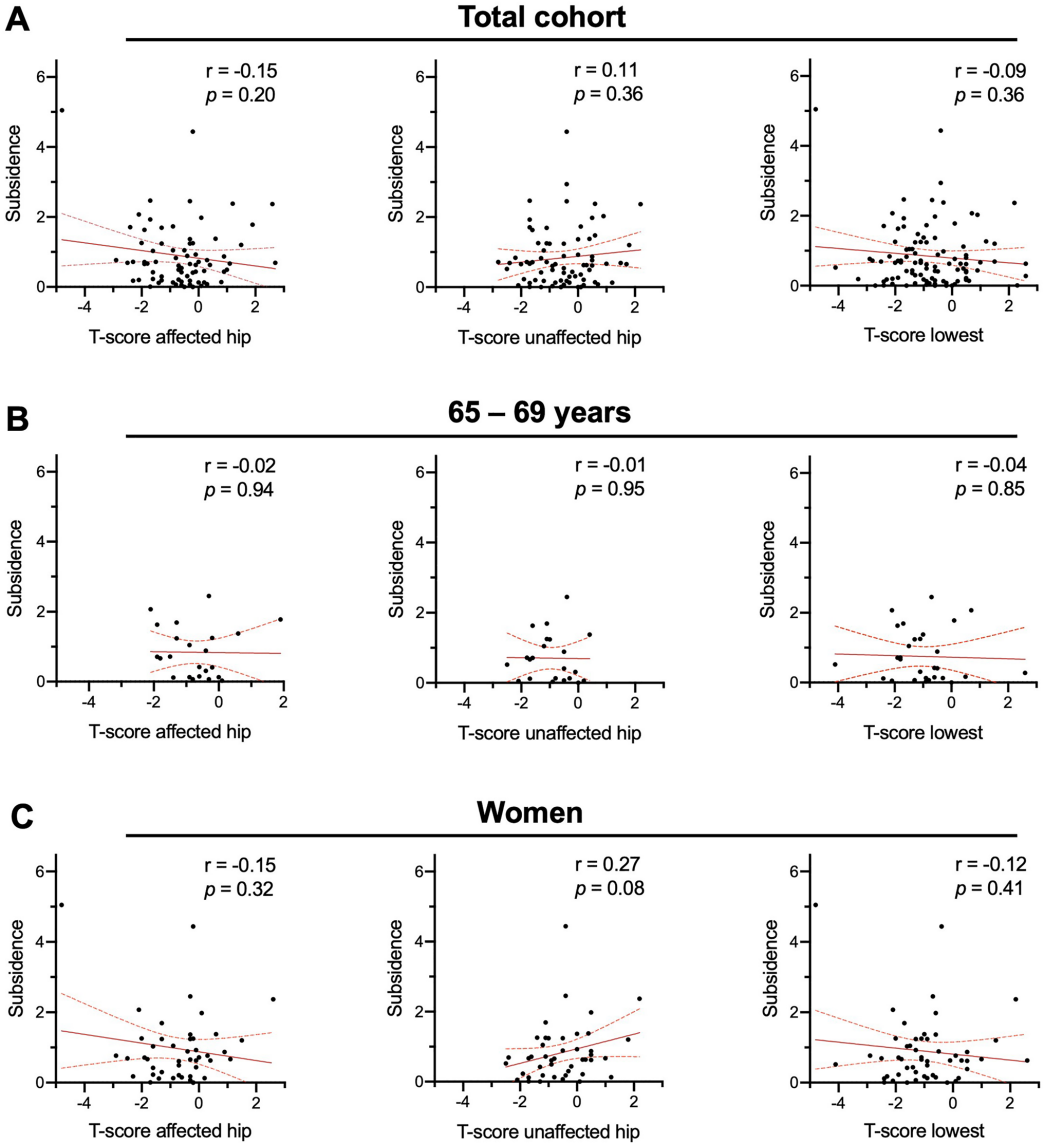
Associations between DXA values and stem subsidence. Correlations between T-score in the affected hip, unaffected hip, and lowest T-score in **A** the total cohort, **B** patients aged 65–69 years (n = 30), and **C** women only (n = 52). Pearson r- and p-values are presented in each panel. Furthermore, 95% confidence bands of the best fit line derived from a simple linear regression analysis are shown

Individual evaluation of the two cases with stem subsidence greater than 3 mm revealed the presence of either morbid obesity (BMI 36.4 kg/m^2^) or severe osteoporosis (T-score − 4.7) (Table 4). However, there were no significant correlations between T-scores and subsidence in the total cohort, neither when using the T-score of the affected hip, unaffected hip, or lowest T-score (Fig. 2A). Moreover, there were also no correlations between T-scores and subsidence in the cohort of patients aged 65–69 years (n = 30, Fig. 2B) or when only women were analyzed (n = 52, Fig. 2C). In the presence of osteoporosis (lowest aBMD T-score ≤ − 2.5), the relative risk of experiencing stem subsidence > 2 mm compared with not having osteoporosis was not significantly increased (1.48; 95% CI: 0.25 to 6.52, p = 0.53).

**Table 4:** Case presentation of the two cases with subsidence > 3 mm

Case	Age	Sex	BMI	T-score lowest	CFI	CFR shoulder	CFR mid	CFR distal	Subsidence (mm)	Dev. initial stem ang. (°)
I	64	f	36.4	− 0.4	3.10	0.867	0.925	0.887	4.4	0.2
II	61	f	19.6	− 4.8	3.34	0.941	0.986	0.795	5.1	− 3.4

*m* male, *f* female, *CFI* canal flare index, *CFR* canal fill ratio, *BMI* body mass index, *Dev* deviation, *ang* angulation

## Discussion

It is well known that reduced BMD in the setting of uncemented THA is associated with an increased risk of potential complications, such as delayed osseointegration [14], intraoperative periprosthetic fracture [15], and aseptic loosening [16]. Regarding femoral stem subsidence, previous studies were limited to small cohorts and showed conflicting findings with both proven [14] and nonexistent [17] associations between DXA aBMD and stem subsidence, depending on the cohort and measurement site. Here, we demonstrated a lower prevalence of decreased aBMD (osteoporosis, osteopenia) in patients younger than 70 years compared to patients older than 70 years, justifying uncemented stem fixation. This cohort of patients < 70 years also showed a low rate of stem subsidence, with subsidence greater than 3 mm occurring in only 2/100 cases. While these cases suggest morbid obesity and severe osteoporosis as individual reasons for subsidence, DXA aBMD values did not correlate with stem subsidence in the total cohort, underlining that low aBMD may not be a relevant risk factor for stem subsidence, at least in a representative cohort of female and male patients < 70 years with low rates of osteoporosis and adequate press fit.

In the cohort of patients younger than 70 years, the frequency of osteoporosis and osteopenia was 7% and 39%, respectively. In contrast, DXA aBMD T-scores were lower in the cohort of patients ≥ 70 years, with 18% and 44% suffering from osteoporosis and osteopenia, respectively. In line with these findings, a high prevalence and undertreatment of osteoporosis in older patients scheduled for THA has been previously demonstrated [3], supporting cemented fixation to prevent bone-related complications in this cohort. Nonetheless, the fact that no age differences could be found in DXA values determined at the lumbar spine highlights a possible positive effect of degeneration and osteophytes on T-score, underscoring the limited informative value of DXA measurements at the lumbar spine in patients with OA [18]. Another reason for why the differences in aBMD T-scores were smaller than expected may be that patients in the young cohort had a higher average body weight, which also reflects obesity as a risk factor especially in young patients with primary OA.

The extent of stem subsidence (mean 0.85 ± 0.88 mm) in our cohort was relatively low compared to other studies but is comparable to a previous study with subsidence of 0.9 + 0.8 mm (anatomic Benoist Girard II (ABG-II) stem, Stryker) [14]. Ries and colleagues analyzed 199 collarless uncemented stems (Corail, DePuy, mean 64.4 years, mean BMI 28.6 kg/m^2^) and observed a mean subsidence of 3.1 ± 2.8 mm [10]. In this previous study, CFR was 80% at the mid stem and 73% at 2 cm above stem tip [10]. In the present study, much higher CFR values were observed on average with 96% and 90%, respectively, indicating sufficient press fit. Ishii and colleagues recommended a CFR of 90% in the region 7 cm distal to the lesser trochanter for optimal press fit [19], which is almost between the two regions measured in the present study.

It is assumed that subsidence around 1 mm is even beneficial for impaction and thus osseointegration, associated with improved clinical outcomes [20]. Since we used clinical radiographs without additional markers (i.e., radiostereometric analysis (RSA)) for subsidence measurements, we set the limit for relevant subsidence at 3 mm to compensate for potential measurement inaccuracies [10]. This cutoff of 3 mm was also previously associated with early failure [11]. While stem subsidence is potentially also influenced by the stem design, the correct stem position and sufficient press fit in our cohort was represented by the neutral stem angulation and the high CFR at all three regions (stem shoulder: 75%; mid stem: 96%; 2 cm above stem tip: 90%). Undersizing of the femoral stem has been associated with early subsidence and aseptic loosening [21]. It has further been demonstrated that a low CFR, especially at the proximal femur, is associated with failed osseointegration [19]. It is therefore likely that the high CFR contributed to the low subsidence rate, ultimately allowing favorable prosthesis survival. In line with these findings, previous studies have shown a 7-year survival rate of 98.9% for the Avenir stem [22].

In our cohort, femoral stem subsidence was not related to femoral anatomy (CFI) and press fit (CFR) as well as demographic factors such as age, sex, and BMI. A previous study also failed to confirm these associations [10]. However, another study was able to identify age and CFI as risk factors for delayed rotational stability [14]. We assume that anatomical factors have an influence on stem subsidence, especially when inadequate press fit or malposition of the stem is present.

We were unable to demonstrate any effect of aBMD T-scores on stem subsidence in our cohort of 100 female and male patients. In a recent retrospective study, patients with osteopenia and osteoporosis also did not demonstrate higher rates of subsidence [23]. In another previous study in 62 patients undergoing uncemented THA (mean age 64 years), aBMD T-scores at the proximal femur were also not associated with stem subsidence as determined by RSA [17]. However, no patients with osteoporosis by DXA (i.e., T-score ≤ − 2.5) were included in this previous study. Another RSA study of postmenopausal women showed that aBMD of the distal radius, but not aBMD of the total hip, was able to discriminate between stem subsidence < 2 mm and ≥ 2 mm [24]. In a study by Aro and colleagues, patients with low systemic aBMD by DXA had greater femoral stem subsidence than patients with normal aBMD. However, this comparison was limited to a small cohort of 39 women [14]. Similarly, intertrochanteric volumetric BMD assessed by quantitative computed tomography in 65 postmenopausal women was associated with stem subsidence [20]. Together, the reasons for the divergent results are likely due to the different measurement methods and sites, as well as the inclusion of different patient cohorts, as we studied women (premenopausal and postmenopausal) and men. Of note, subgroup analysis also showed no correlations between aBMD T-score and subsidence when only patients between 65 and 69 years of age or only women were evaluated. Nonetheless, supported by the results of two cases with stem subsidence, cemented fixation should be considered also in patients < 70 years with morbid obesity and severe osteoporosis. Given the limitations of DXA, future studies should focus on three-dimensional high-resolution techniques for measuring bone quality.

A limitation of our study is that the data were analyzed retrospectively, which prevents conclusions about THA survival in relation to stem subsidence. The study design also did not allow for uniform time points of the follow-up measurements, although a minimum follow-up of eight weeks after surgery was guaranteed in line with a previous study [10]. Previous studies have shown that subsidence occurs mainly in the first 6 weeks [25]. Another limitation of our study is that evaluation was based on clinical radiographs, whereas previous RSA studies have used radiopaque markers to analyze stem subsidence. While RSA may be more accurate, radiological subsidence measurements were performed on standardized radiographs according to a previous study [10], enabling transferability in the clinical context. A further limitation of our study was that we performed a DXA examination, which, although the gold standard for detecting osteoporosis, is unable to account for bone microarchitecture and results in artificially high values at measurement sites affected by OA. Finally, while this is the largest cohort combining DXA and subsidence analysis in a representative cohort of both women and men, the reason for the lack of associations between DXA values and stem subsidence could also be due to the inclusion of both sexes, the low rate of subsidence as well as the low rate of osteoporosis. Low aBMD value could be a more relevant risk factor in case of suboptimal stem position.

## Conclusion

Our study suggests that low aBMD assessed by DXA does not appear to be associated with the risk of stem subsidence in patients younger than 70 years with adequate press fit. However, low aBMD and morbid obesity emerged as potential contributing factors in two cases with relevant subsidence. The impact of bone quality on stem subsidence should be investigated in future studies, preferably in high-risk patient cohorts and using advanced imaging techniques beyond DXA.

## Data Availability

The data that support the findings of this study are available from the corresponding author upon reasonable request.
